# Evaluation of 3D-Printed Models in Dental Education: Practical Applications and Student Perspectives

**DOI:** 10.7759/cureus.81644

**Published:** 2025-04-03

**Authors:** Todor G Bogdanov, Todor A Hikov, Todor Uzunov, Dimitar Kirov, Nikola Milanov, Hristina Galeva

**Affiliations:** 1 Medical Physics and Biophysics, Faculty of Medicine, Medical University - Sofia, Sofia, BGR; 2 Prosthetic Dental Medicine, Faculty of Dental Medicine, Medical University - Sofia, Sofia, BGR

**Keywords:** 3d printing, cad/cam technology, dental education, digital dentistry, prosthetic training, student perception

## Abstract

Introduction: Recently, developments in digital technologies have significantly transformed dental education and practice. This study aims to assess students' knowledge and interest in digital dentistry and evaluate the implementation of 3D-printed models in dental training. A survey was conducted among 249 dental students to gauge their understanding of digital technologies, their willingness to deepen their knowledge, and their perceptions of advantages and limitations compared to conventional methods.

Materials and methods: Following the survey, 3D-printed dental models with removable dies were fabricated using high-resolution resin printing technology and distributed among first-year dental students. Over 13 weeks, students utilized these models for prosthetic training, performing procedures such as wax modeling, casting, and polishing of dental crowns. The study assessed the models’ durability and categorized defects based on their location and cause.

Results: The results revealed a high level of student interest in digital technologies, emphasizing the necessity of integrating modern tools into dental education. Statistical analysis of the defects in the 3D-printed models highlighted critical areas requiring improvement, particularly concerning mechanical handling. Additionally, correlation analyses of student responses indicated that those who perceive digital tools as beneficial for their future careers are more inclined to adopt them in practice.

Conclusions: The findings underscore the importance of digitalization in dental training, highlighting both its benefits and areas for refinement. Enhancing processing techniques and optimizing training methodologies can further improve the quality of 3D-printed models and their effectiveness in dental education.

## Introduction

In the age of digital technologies, they are an essential part of the working methods in medicine, including dentistry [1-4]. The benefits of their use are undeniable - saving time and materials, better visualization of expected results, better communication with patients [5], more predictable results and the ability to make adjustments fast and easy when necessary [6].

The specifics of work, the great variety and the constant renewal of technologies and materials used in the field of dental medicine bring with them the expected need for training of dental specialists [7-9]. Studies have been conducted on their opinions on this need. Most dental practitioners find this type of education useful and necessary so that they can propose to the patients contemporary dentistry [10,11].

Digital dentistry completely changes daily dental practice. For that reason, some universities make similar investigations about students' opinions [12]. Most students find it essential to have enough knowledge and skills to use digital technologies for their future practice. Their results also confirm the need to educate students in the use of modern digital technologies in dentistry [13]. As a result, different digital technologies are implemented in the student's education, such as computer-aided design (CAD) and computer-aided manufacturing (CAM) technologies, intraoral scanners, additive technologies, and artificial intelligence [14-16].

3D-printed models are used in surgical training, implantology, and prosthetic and root canal treatment [17]. This kind of model allows both to recreate the anatomy and to recreate it according to the individual specifics of different patients. This makes them extremely valuable in the training of both students and practitioners, especially in the fields of surgery, implantology and prosthetics [18,19].

The aim of this study is to explore students’ knowledge regarding the application of digital technologies and 3D printing in dentistry, their interest in acquiring deeper expertise in the field, and the potential integration of such technologies into their educational training.

## Materials and methods

To evaluate the awareness and understanding of digital technologies in dentistry, a structured survey was administered to 249 second-year students enrolled in the Dental Medicine program at the Faculty of Dental Medicine, Medical University - Sofia, Sofia, Bulgaria. The questions included their interest in studying the application of these technologies more deeply, as well as the advantages and disadvantages they found when comparing them with conventional methods of work.

After analyzing the results of the survey, dental working models with removable dies for the upper and the lower jaw were made by 3D printing technology. For the maxilla, three removable dies were made, and two for the mandibula. In the fabrication of 3D-printed models with removable dies, a Phrozen Mighty 8K (Phrozen Tech Co., Ltd., Hsinchu City, Taiwan) resin printer was utilized. This advanced printer features a 10-inch LCD screen and operates with a 405 nm wavelength for polymerization. The model production relied on Anycubic Basic resin (Shenzhen Anycubic Technology Co., Ltd., Shenzhen, Guangdong, People's Republic of China), to which CMYK Pigments from FEPshop (FEPshop Ltd., Groningen, Netherlands) were added for color customization. This combination allowed for the precise creation of detailed dental models suitable for clinical applications. The printing parameters were meticulously set to ensure high resolution and accuracy. A layer thickness of 0.05 mm was used, which provided fine detail for each print layer. The exposure was set to 0.5 seconds to optimize the curing process, preventing overexposure. For the base layers, which are critical for model adhesion and stability, the bottom exposure time was extended to 25 seconds with five bottom layers to ensure a strong foundation. Additional print settings included a Z lift distance of 8 mm, with both the Z lift speed and Z retract speed set at 6 mm/s, which facilitated smooth layer transitions and minimized any risk of print defects. Anti-Aliasing Level 1 was applied to reduce edge distortions, further enhancing the overall model precision. All slicing and preparation tasks were performed using CHITUBOX (Shenzhen Anycubic Technology Co., Ltd.), a reliable slicer known for its compatibility with resin printers and its ability to optimize print settings for intricate models like those used in dental applications. This combination of materials, advanced printer technology, and precise settings successfully created highly detailed 3D-printed dental models with removable dies, suitable for educational purposes.

**Figure 1:**
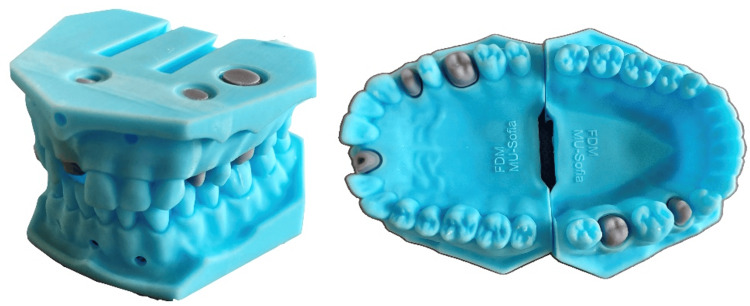
3D printed models

A total of 230 pairs of these working models, presented in Figure 1, were provided to first-year students in the Faculty of Medicine, Medical University - Sofia. They used them to make one post-core for the upper central incisor and five full crowns (three metal crowns, one metal-resin crown and one temporary resin crown).

**Figure 2:**
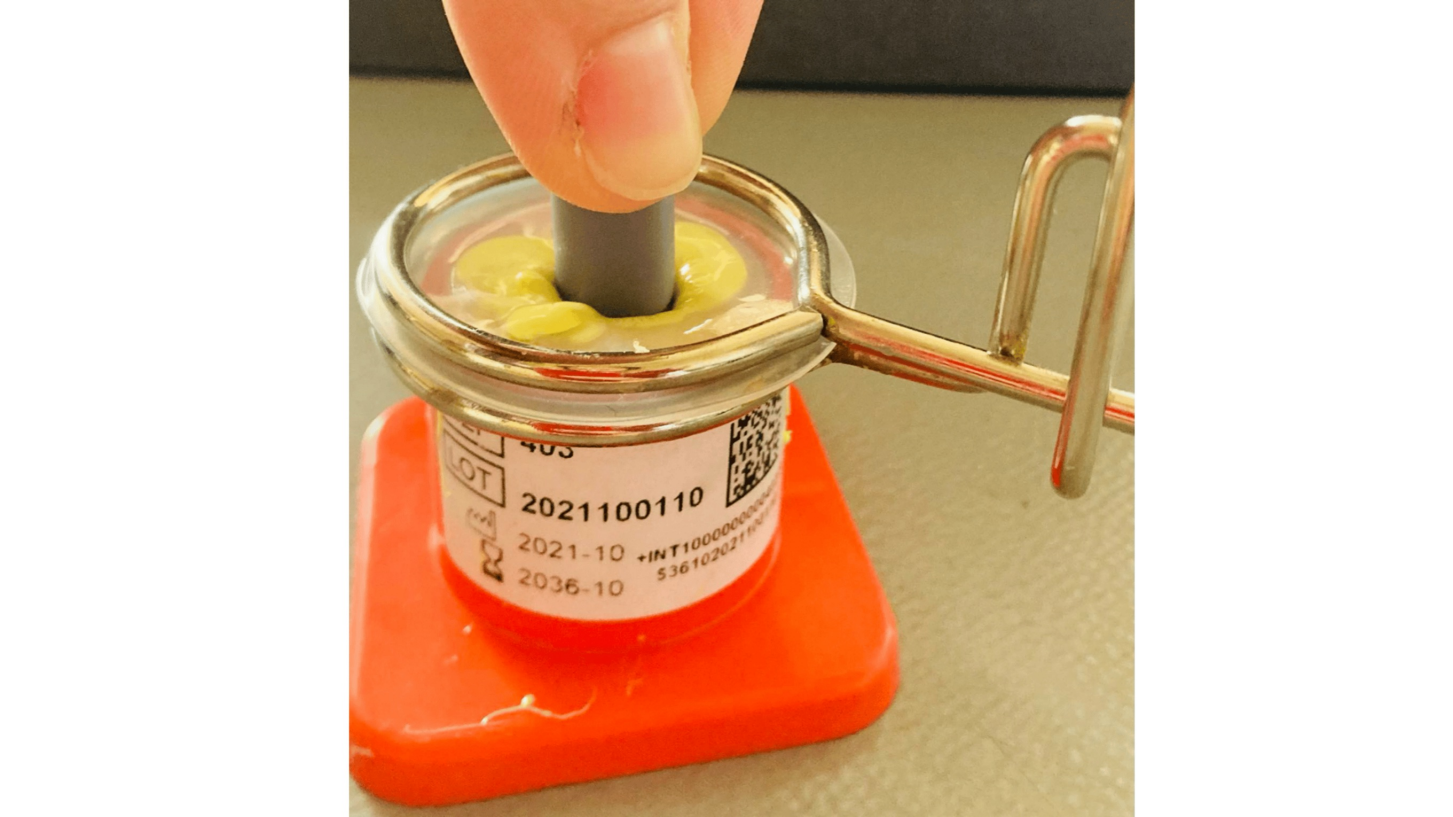
Shaping Adapta foil

The models were fixed in articulator with second-class gypsum material. The crowns for the upper jaws were made with the Adapta system (BEGO GmbH & Co. KG, Bremen, Germany). The base and spacer foil were heated on the flame of an alcohol lamp, positioned on the top of the silicone vanish, and the 3D printed dies pressed into the foil (Figure 2).

**Figure 3:**
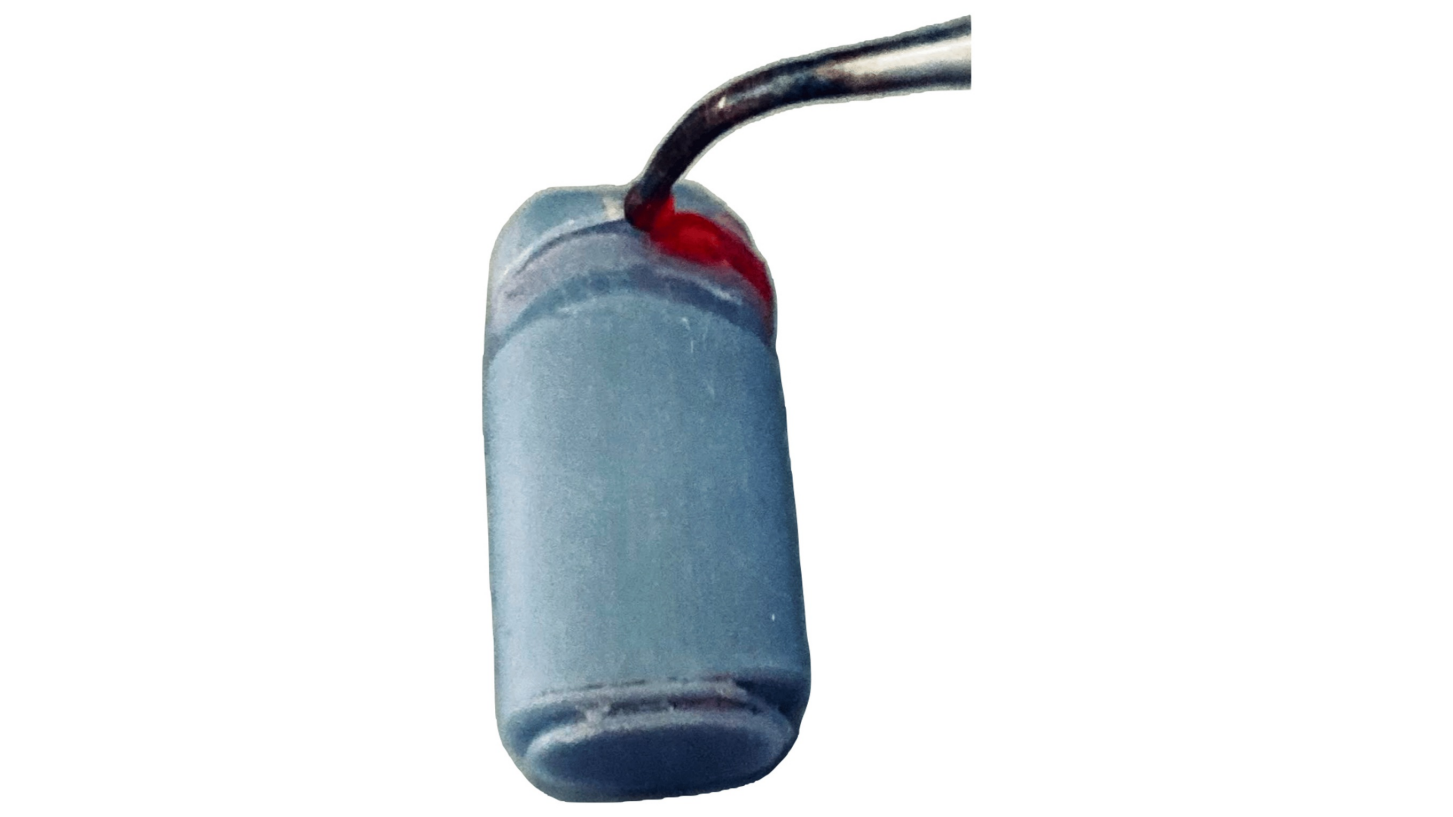
Application of cervical wax

The foil was cut by the students in 0.5 mm up to the marginal design of the die, isolated with vaseline, and cervical wax was applied directly (Figure 3).

**Figure 4:**
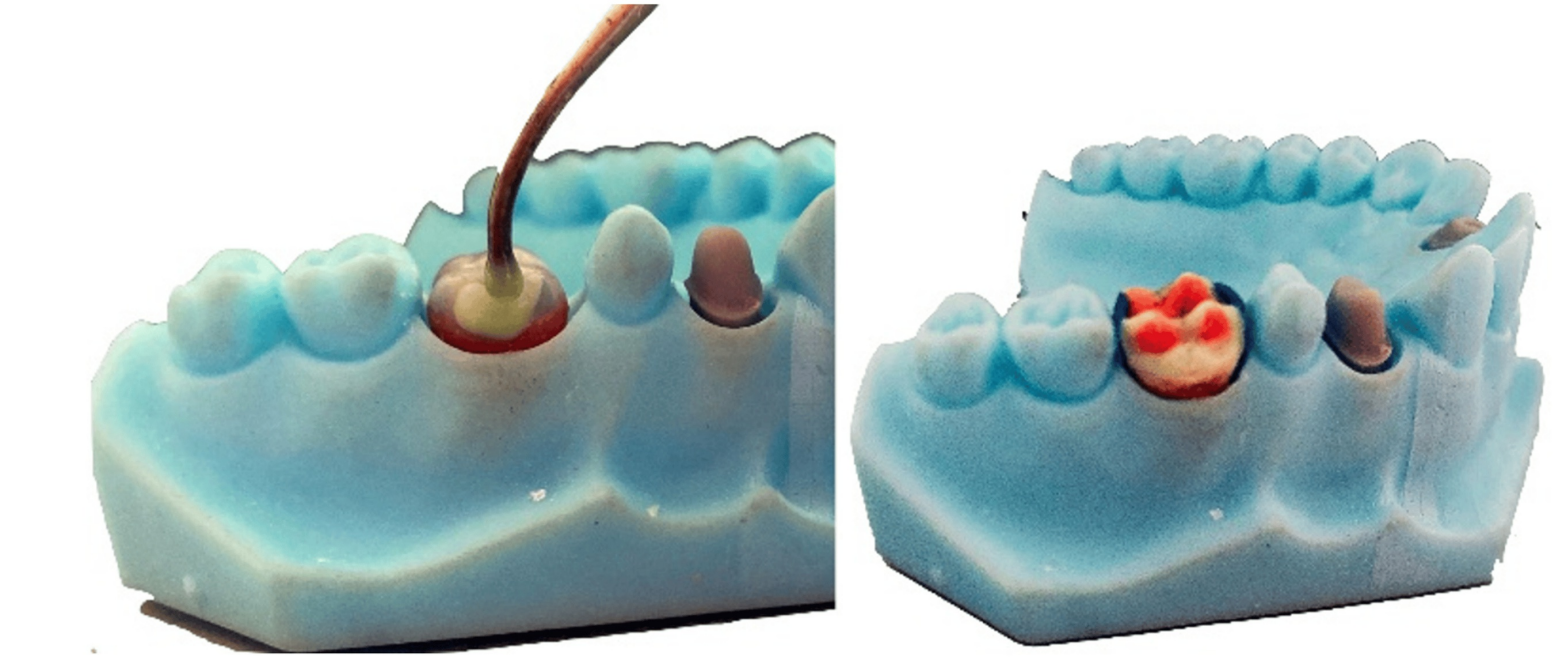
Wax modeling - Thomas' technique

The students created wax models of the crowns using Thomas’ technique (Figure 4).

For the lower jaw crowns, the wax dipping technique was employed. In this case, the isolated lower dies were put in a special vanish full of melted wax (melting temperature: 96°C) for two to three seconds. The additional wax was then manually removed by the students. Following the same approach as the upper jaw crown preparation, 0.5 mm of wax up to the marginal design was carefully reduced. After isolating the die, cervical wax was applied by the students. The wax modeling of the crowns was executed using Thomas’ technique.

**Figure 5:**
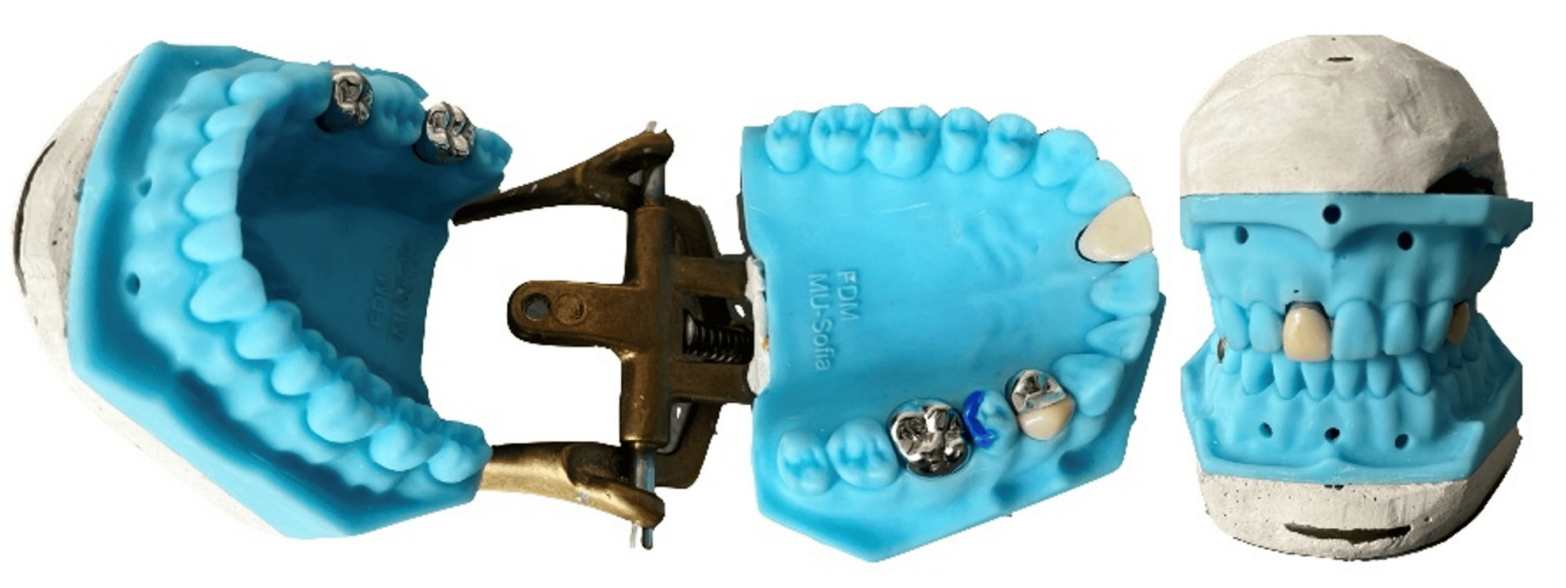
Adjusted and polished crowns

Dental technicians used the wax prototypes to cast metal crowns using Cristalloy-M (Co-Cr-Mo dental alloy) and Wirovest (investment material). After the casting, the metal crowns were adjusted to the removable dies by the students. They used occlusal spray to check the fitting to the crowns. Occlusal contacts were checked with articulation paper and reduced where it was necessary. The crowns were polished by the students in the following order: stones, rubbers, and brushes, as shown in Figure 5.

**Figure 6:**
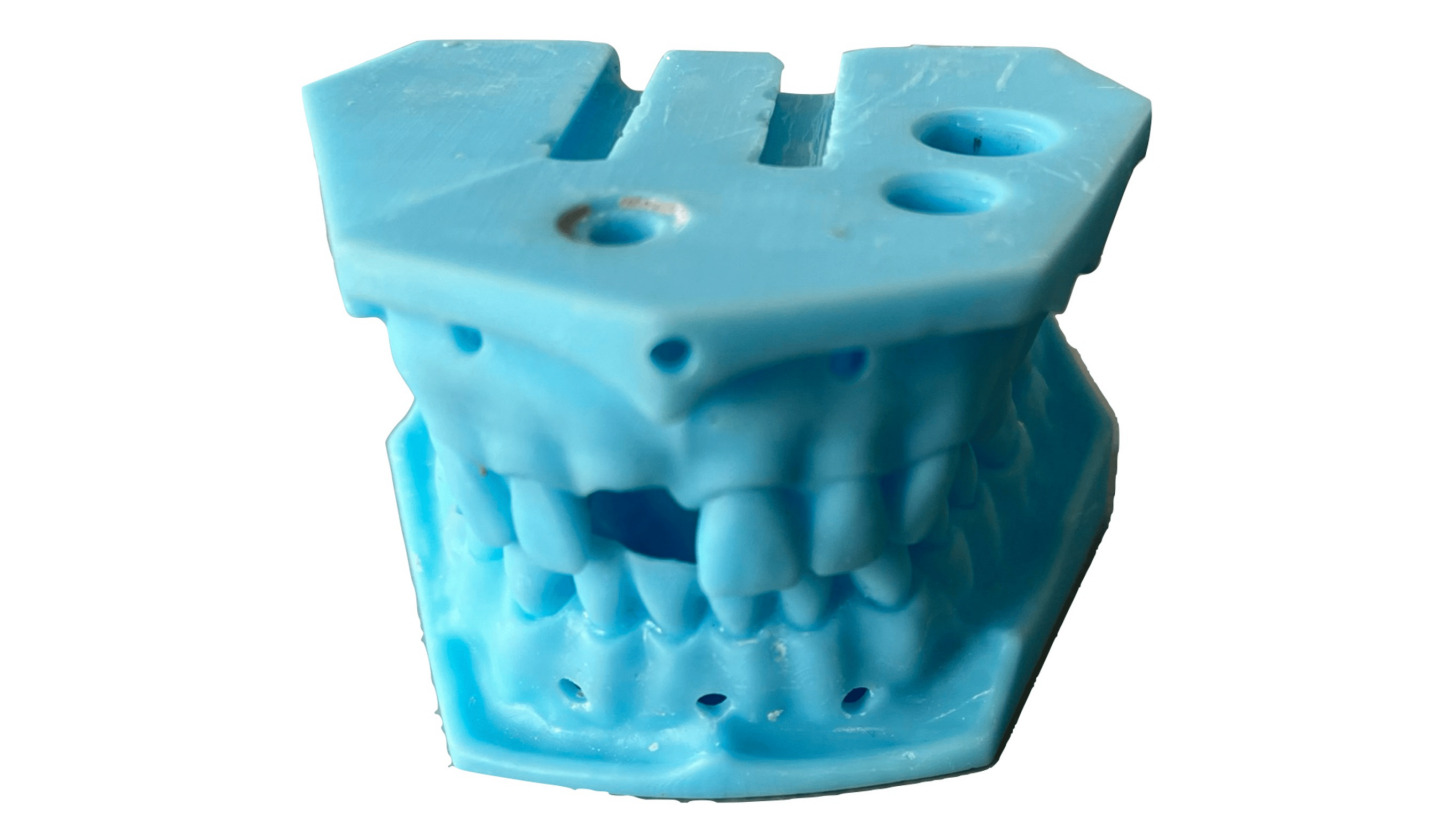
Defects out of the prosthetic field

**Figure 7:**
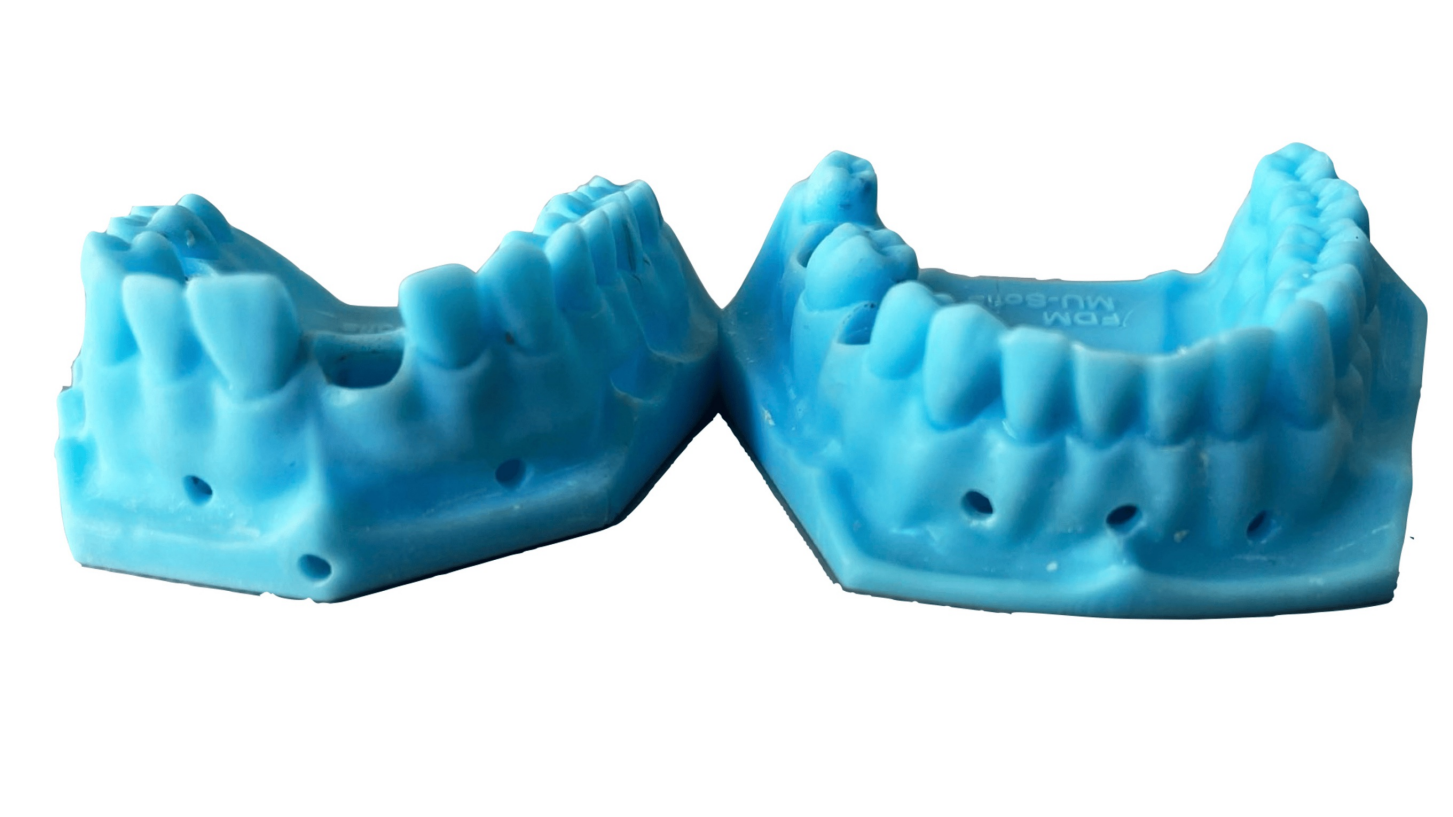
Defects in the area of interest

**Figure 8:**
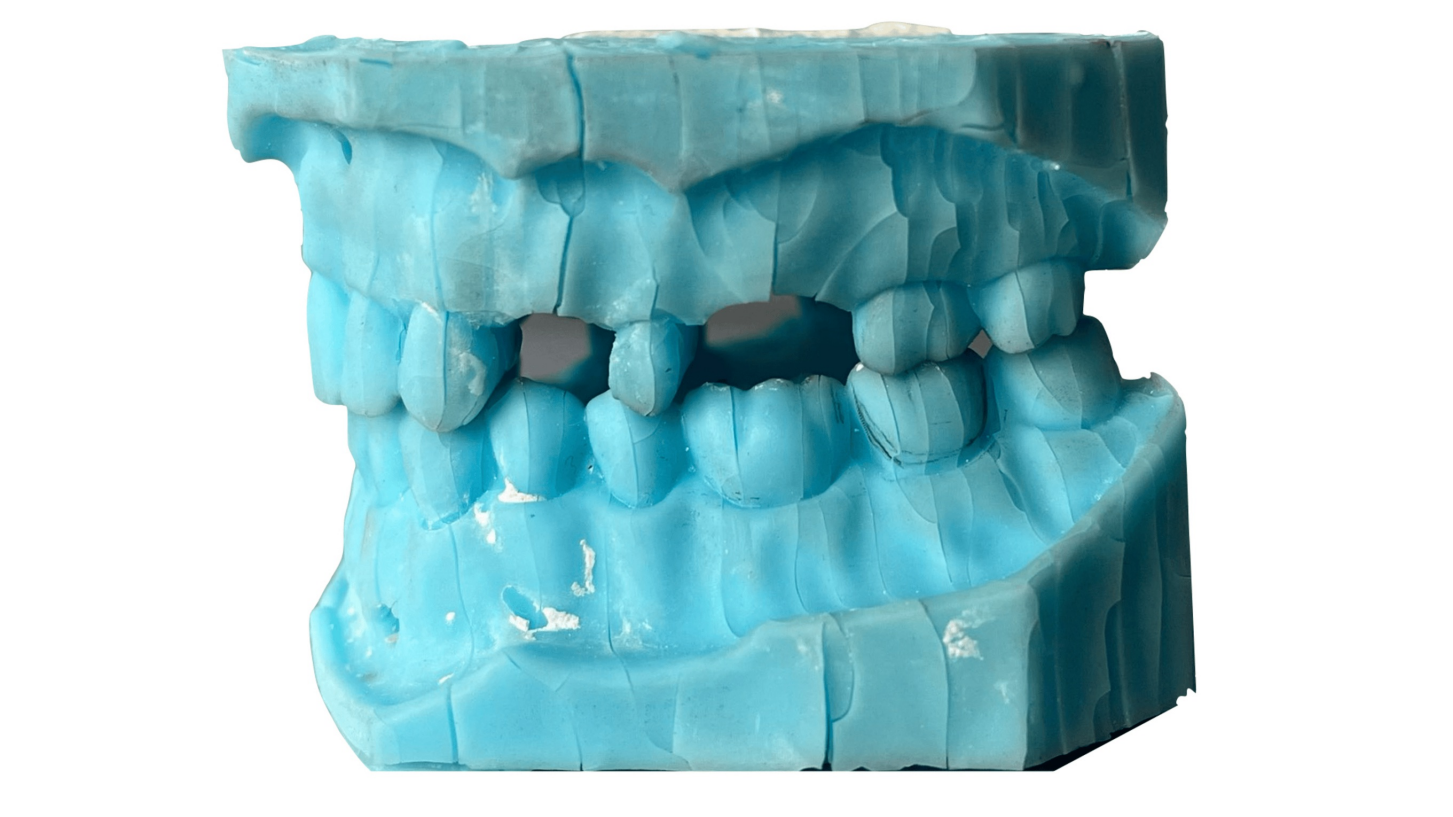
Defects in the prosthetic field

**Figure 9:**
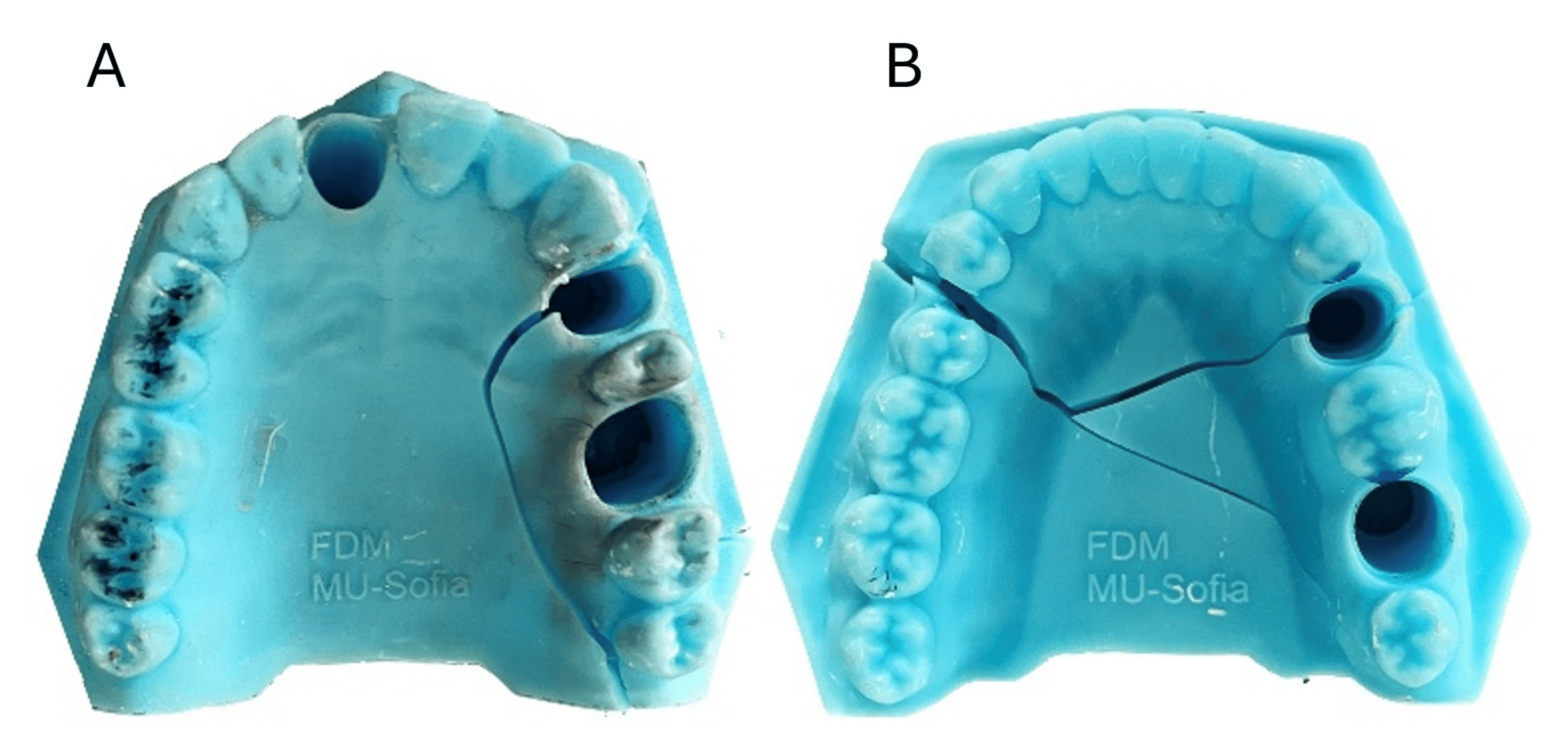
3D printed models damaged by external loads for upper jaw (A) and lower jaw (B)

Students worked with these models for 13 weeks. Finally, they removed the 3D-printed models from the articulators. The models' condition was then reviewed. According to the damage established, the final state was divined in five groups: group 1 - without defects, group 2 - with defects out of the prosthetic field (see Figure 6), group 3 - with defect in the area of interest (see Figure 7), group 4 - with defect in the prosthetic field (see Figure 8) and group 5 - damaged by external loads/during removing by the articulator (see Figure 9).

## Results

Defects in the samples

Table 1 presents the results for the number of defects in each group for the corresponding samples. The number of defect-free samples has been counted, along with the number of defects in each of the following groups: defects outside the working area, defects in the region of interest, defects in the working area, and defects due to external forces. In some samples, defects were observed in only one group, while in others, defects were present in two or more groups simultaneously. The summarized results are presented in Table 1.

**Table 1:** Defects distribution

	Bottom samples			Upper samples		
	Total number of deffects	Samples per group	Average defects per group	Total number of deffects	Samples per group	Average defects per group
No defects	55	55	1	47	47	1
Defects outside the working area	231	120	1.93	211	113	1.87
Defects in region of interests	32	21	1.52	25	17	1.47
Defects in the working area	210	122	1.72	245	134	1.83
Defects due to external forces	205	111	1.85	233	122	1.91

**Figure 10:**
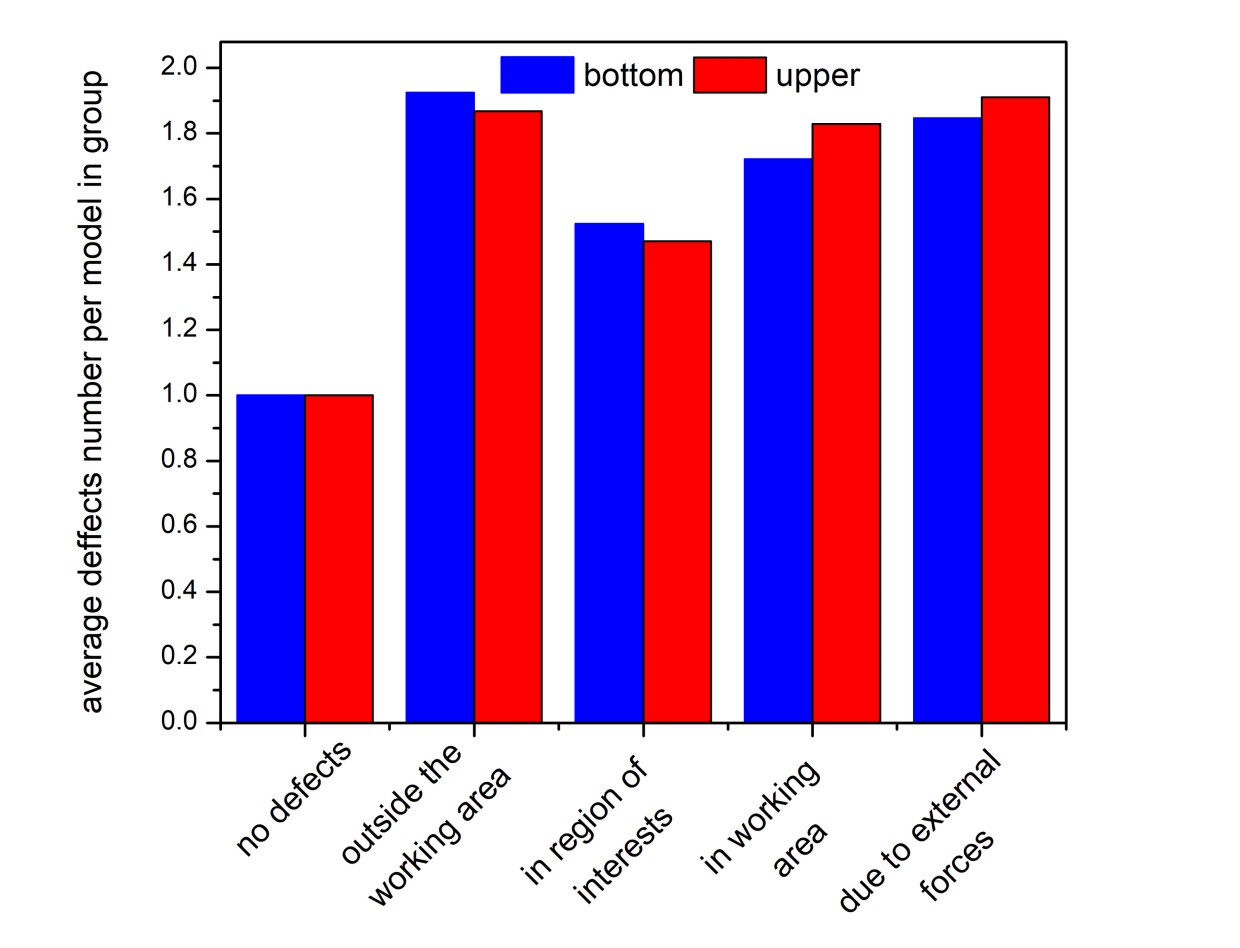
Average number of defects in different categories for lower jaw samples (blue) and upper jaw samples (red)

Figure 10 presents the average number of defects in different categories for lower jaw samples (blue) and upper jaw samples (red).

For both groups, 55 lower jaw samples and 47 upper jaw samples were observed without damage. The results suggest a certain degree of similarity between the two sets of samples. These preliminary observations provide a broader perspective, which warrants a more in-depth statistical analysis of the results. By performing p-value calculations using Welch’s t-test for independent samples, aimed at comparing the mean values of two independent groups (in this case, defects in the upper and lower models), we obtain the results presented in Table 2.

**Table 2:** Bottom-upper samples’ defects correlation

p-value for bottom-upper samples correlation	
Defects type	p-value
Outside the working area	0.60077
In region of interests	0.77915
In the working area	0.29703
Due to external forces	0.73551

In this case, the p-values are relatively high (all > 0.05), indicating that there is no statistically significant difference in the number of defects between the upper and lower models for any of the categories. As a result, a separate analysis was conducted to examine the relationships between defects within the lower and upper samples.

**Figure 11:**
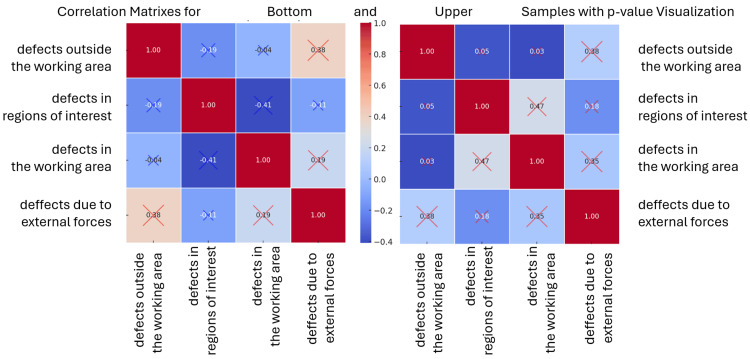
Pearson correlation coefficients (r) in correlation matrices for lower (left) and upper (right) samples, with corresponding p-values

Figure 11 presents the Pearson correlation coefficients (r) in correlation matrices for the lower and upper samples, along with the corresponding p-values represented as circles. These coefficients measure the linear relationship between each pair of defect categories. Numerical data is presented in Table 3.

**Table 3:** Correlation coefficients (r), t-values and p-values

	Upper samples											
	Defects outside the working area			Defects in region of interests			Defects in the working area			Defects due to external forces		
	r	t-value	p-value	r	t-value	p-value	r	t-value	p-value	r	t-value	p-value
Defects outside the working area	1	-	-	0.05	0.265	0.89060	0.03	0.159	0.80416	0.38	2.174	0.00017
Defects in region of interests	0.05	0.265	0.89060	1	-	-	0.472	2.833	0.19906	0.184	0.991	0.63644
Defects in the working area	0.03	0.159	0.80416	0.472	2.833	0.19906	1	-	-	0.355	2.009	0.00045
Defects due to external forces	0.38	2.174	0.00017	0.184	0.991	0.63644	0.355	2.009	0.00045	1	-	-
	Bottom samples											
	Defects outside the working area			Defects in region of interests			Defects in the working area			Defects due to external forces		
	r	t-value	p-value	r	t-value	p-value	r	t-value	p-value	r	t-value	p-value
Defects outside the working area	1	-	-	-0.19	-0.996	0.56485	-0.04	-0.191	0.75790	0.385	2.207	0.00010
Defects in region of interests	-0.19	-0.996	0.56485	1	-	-	-0.41	-2.365	0.24150	-0.12	-0.613	0.76887
Defects in the working area	-0.04	-0.191	0.75790	-0.41	-2.365	0.24150	1	-	-	0.189	1.860	0.08345
Defects due to external forces	0.385	2.207	0.00010	-0.12	-0.613	0.76887	0.189	1.860	0.08345	1	-	-

When analyzing the correlation dependencies between different types of defects in the upper and lower models, both similarities and significant differences are observed. These relationships reveal how defects in one area may be linked to other issues in different parts of the models, as well as how external factors, such as mechanical forces applied to the models, influence the overall quality of the final results.

Student opinions

A survey was conducted among 106 students who worked with the samples, consisting of 10 questions regarding their opinions on digital methods in dental medicine, including the use of 3D-printed models and virtual imaging. The questionnaire is included in Appendix A to this article, along with a table containing the responses of each student. Table 4 presents a summary of the number of responses for each question.

**Table 4:** Distribution of questionnaire answers

Answer/Question	Q1	Q2	Q3	Q4	Q5	Q6	Q7	Q8	Q9	Q10
A	82	95	85	29	87	89	83	40	62	85
B	15	11	21	58	4	6	5	34	7	3
C (if applicable)	9	0	0	15	14	10	18	25	28	17
D (if applicable)	0	0	0	0	0	0	0	0	8	0

**Figure 12:**
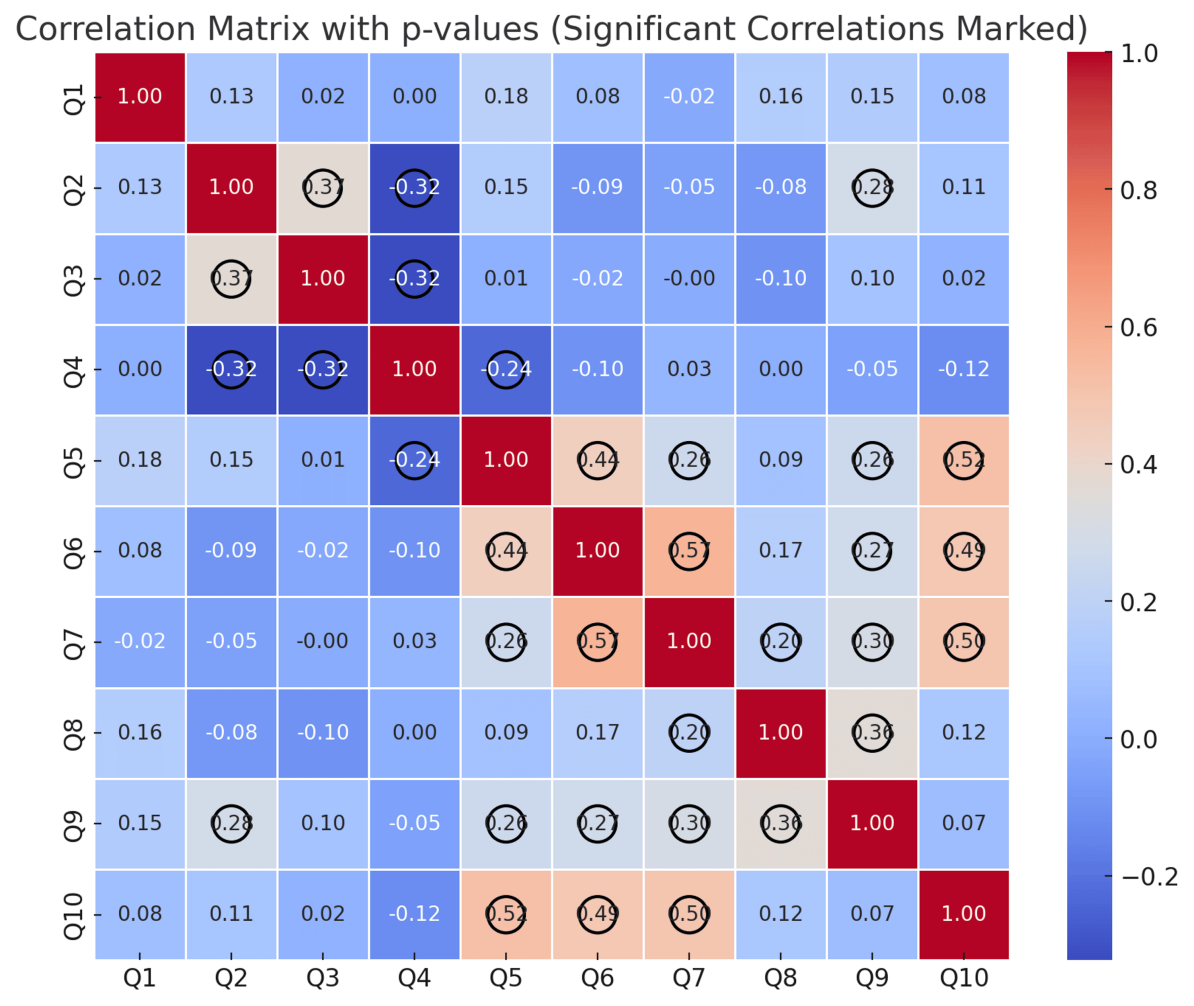
Pearson correlation coefficients (r) and related p-values for the students' responses

The Pearson correlation coefficients (r) and their corresponding p-values were calculated for the students' responses. The results are presented graphically in Figure 12 and tabulated in Table 5.

**Table 5:** The correlation coefficients (r), related t-values and p-values for the students' responses

	Q1			Q2			Q3			Q4			Q5			Q6			Q7			Q8			Q9			Q10		
	r	t-value	p-value	r	t-value	p-value	r	t-value	p-value	r	t-value	p-value	r	t-value	p-value	r	t-value	p-value	r	t-value	p-value	r	t-value	p-value	r	t-value	p-value	r	t-value	p-value
Q1	1	-	-	0.13	1.34	0.299	0.02	0.2	0.815	0	0	0.879	0.18	1.87	0.056	0.08	0.82	0.395	-0.02	-0.2	0.919	0.16	1.65	0.080	0.15	1.55	0.110	0.08	0.82	0.434
Q2	0.13	1.34	0.299	1	-	-	0.37	4.06	0.000	-0.32	-3.44	0.000	0.15	1.55	0.080	-0.09	-0.92	0.389	-0.05	-0.51	0.696	-0.08	-0.82	0.593	0.28	2.97	0.001	0.11	1.13	0.272
Q3	0.02	0.2	0.815	0.37	4.06	0.000	1	-	-	-0.32	-3.44	0.001	0.01	0.1	0.946	-0.02	-0.2	0.820	0	0	0.950	-0.1	-1.02	0.288	0.1	1.02	0.342	0.02	0.2	0.846
Q4	0	0	0.879	-0.32	-3.44	0.000	-0.32	-3.44	0.001	1	-	-	-0.24	-2.52	0.017	-0.1	-1.02	0.350	0.03	0.31	0.660	0	0	0.822	-0.05	-0.51	0.714	-0.12	-1.23	0.227
Q5	0.18	1.87	0.056	0.15	1.55	0.080	0.01	0.1	0.946	-0.24	-2.52	0.017	1	-	-	0.44	5	0.000	0.26	2.75	0.008	0.09	0.92	0.382	0.26	2.75	0.009	0.52	6.21	0.000
Q6	0.08	0.82	0.395	-0.09	-0.92	0.389	-0.02	-0.2	0.820	-0.1	-1.02	0.350	0.44	5	0.000	1	-	-	0.57	7.07	0.000	0.17	1.76	0.099	0.27	2.86	0.005	0.49	5.73	0.000
Q7	-0.02	-0.2	0.919	-0.05	-0.51	0.696	0	0	0.950	0.03	0.31	0.660	0.26	2.75	0.008	0.57	7.07	0.000	1	-	-	0.2	2.08	0.047	0.3	3.21	0.002	0.5	5.89	0.000
Q8	0.16	1.65	0.080	-0.08	-0.82	0.593	-0.1	-1.02	0.288	0	0	0.822	0.09	0.92	0.382	0.17	1.76	0.099	0.2	2.08	0.047	1	-	-	0.36	3.94	0.000	0.12	1.23	0.235
Q9	0.15	1.55	0.110	0.28	2.97	0.001	0.1	1.02	0.342	-0.05	-0.51	0.714	0.26	2.75	0.009	0.27	2.86	0.005	0.3	3.21	0.002	0.36	3.94	0.000	1	-	-	0.07	0.72	0.492
Q10	0.08	0.82	0.434	0.11	1.13	0.272	0.02	0.2	0.846	-0.12	-1.23	0.227	0.52	6.21	0.000	0.49	5.73	0.000	0.5	5.89	0.000	0.12	1.23	0.235	0.07	0.72	0.492	1	-	-

The correlation analysis of students' responses provides valuable insights into how they perceive digital methods in dental medicine, as well as the relationships between different aspects of their education. The data reveals several key associations that highlight patterns in the opinions of the respondents.

## Discussion

Defects in the samples

When analyzing defects in the upper and lower models, a clear trend emerges: the highest number of samples contain deformations in the working area. This observation is valid for both types of models, with 134 upper samples and 122 lower samples exhibiting imperfections specifically in this region. The working area is the most critical part of the model, where the most precise corrections are made, which likely increases the risk of errors. The high frequency of errors in this zone suggests that it requires special attention during processing and that improvements in technique could reduce the likelihood of errors.

Interestingly, despite the working area having the highest number of affected samples, the total number of impairments in some cases is slightly higher in other categories. For example, in the lower models, the highest absolute number of defects is observed outside the working area (231 defects), whereas 210 defects were recorded in the working area itself. This indicates that while impairments in the working area are the most widespread among the samples, in quantitative terms, other areas may be even more affected. In the upper models, the number of deformations in the working area (245) is slightly higher than that outside of it (211), but the difference is minimal. These variations suggest that different factors may influence the distribution of defects. In some cases, this could be due to specific technical challenges, such as the complexity of the processed surface or the mechanical stresses applied to it. In other cases, factors such as model manipulation during handling and final processing may contribute to the shifting of deformations to other zones.

Another important aspect of the analysis is the impact of external forces on defect formation. Defects caused by external forces - such as removing the model from the articulator - represent one of the most affected categories in both upper and lower models. A total of 205 damages were recorded in the lower models and 233 in the upper models, which are relatively close values. Interestingly, this deformation category has the second-highest frequency, suggesting that mechanical stresses applied to the model may play a significant role in its overall quality.

Another important observation relates to impairments in the region of interest. Although this category of defects is found in a smaller number of samples compared to the working area and damages caused by external forces (21 samples in the lower models and 17 in the upper models), the average number of deteriorations per sample is comparable to other categories (1.52 for the lower models and 1.47 for the upper models). This suggests that while defects in this zone are less frequent, when they do occur, they are likely to be severe and critically impact the quality of the result.

One of the key findings is that defects in the working area show the strongest correlation with defects caused by external forces, a pattern that holds true for both upper and lower models. The correlation between these two factors is moderate (r ≈ 0.35) and statistically significant, suggesting that the way models are processed and handled may have a decisive impact on the final outcome. The removal from the articulator, placement in the working environment, and final mechanical processing are likely to affect the overall quality, leading to an increase in defects in the working area.

Interestingly, external forces are linked not only to defects in the working area but also to those outside it. The correlation between these two categories is also moderate (r ≈ 0.38), indicating that some of the defects occurring outside the working area may not be the result of the actual work on the model but rather how it is manipulated. This highlights the need for controlled processing conditions, particularly during the stages of mechanical handling of the models.

While these relationships are consistent across both types of models, one of the most significant differences between the upper and lower models is observed in the correlation between defects in the region of interest and defects in the working area. In the upper models, defects in the region of interest are often accompanied by defects in the working area (r ≈ 0.47). This suggests that errors in these two areas likely share a common source and may stem from more complex issues related to working techniques or model construction.

In contrast, an inverse relationship is observed in the lower models; defects in the working area and the region of interest are negatively correlated (r ≈ -0.41). This implies that if one of these areas is compromised, the other may remain unaffected. A possible explanation for this phenomenon is that, during the processing of lower models, students tend to focus their attention on one of the two areas - greater precision in the region of interest may lead to fewer defects in the working area and vice versa. This behavioral difference between upper and lower models indicates that in upper models, defects tend to spread across multiple areas, whereas in lower models, problems are more concentrated and localized.

Another difference between the two types of models is related to defects caused by external forces and their influence on the region of interest. In the upper models, this relationship is slightly positive (r ≈ 0.18), whereas in the lower models, there is almost no correlation (r ≈ -0.11). This may indicate that in the upper models, defects resulting from mechanical impact have a broader influence, affecting not only the working area but also the region of interest, while in the lower models, these errors remain more localized.

The statistical significance analysis (p-values) of the correlations confirms that for both types of models, the most significant dependencies exist between defects in the working area and those caused by external forces. This further supports the hypothesis that mechanical impact on the models plays a key role in defect formation. Although some other correlations were also identified, their higher p-values suggest that these relationships may be due to random factors.

The primary difference between the upper and lower models lies in the distribution and spread of defects. In the upper models, defects are more interconnected - if an error occurs in one area, the likelihood of defects appearing in another area is higher. In contrast, defects in the lower models tend to be more localized, which may be a result of how attention is directed toward processing specific zones. In both cases, external forces remain a key factor influencing quality, suggesting that improvements in handling and processing techniques could lead to a significant reduction in defects.

Student opinions

Firstly, a significant positive correlation was found between the perception of 3D-printed models as a modern method (Q2) and the lack of difficulties when working with them (Q3) (r = 0.37, p < 0.001). This suggests that students who consider this technology modern are less likely to experience challenges when using it. At the same time, a negative correlation was observed between the perception of 3D-printed models as contemporary and the preferred working method (Q2 and Q4: r = -0.32, p < 0.001), indicating that those who support digital technologies are more likely to choose working with them over traditional gypsum models.

Another important aspect of the analysis is the relationship between the belief that digital methods will be beneficial for future professional development (Q5) and students' interest in learning about them (Q6) (r = 0.44, p < 0.001). This association underscores the importance of perceiving digital technologies as a competitive advantage in dental medicine, which contributes to a stronger interest among students in acquiring these skills. An even stronger correlation was observed between the perceived usefulness of digital methods and the opinion that 3D virtual images would enhance the understanding of educational material (Q5 and Q10: r = 0.52, p < 0.001). This suggests that students who recognize the practical value of digitalization are also more likely to support the use of interactive visual technologies in their learning process.

An interesting result is also observed in relation to overall satisfaction with the educational process. Students who are satisfied with the knowledge and skills they have acquired (Q9) are more likely to perceive 3D-printed models as a modern method (Q2) (r = 0.28, p < 0.001). This suggests that the integration of digital technologies into education may play a role in fostering a more positive perception of the learning experience.

In conclusion, the results of the correlation analysis indicate that students who support digital technologies find them more useful and prefer working with them. Conversely, those who experience difficulties with digital methods tend to adhere to traditional forms of training. Furthermore, interest in learning about digital technologies in dental medicine is directly linked to the belief that they will contribute to better professional development opportunities for students. These observed relationships emphasize the importance of modern technologies in education and their role in shaping future specialists in the field of dental medicine.

## Conclusions

The present study provides valuable insights into the influence of various factors on the formation of defects in 3D-printed dental models and the perception of digital technologies among dental students. Damage analysis revealed that the working area is the most critical zone, showing the highest frequency of errors. A strong correlation between defects in the working area and those caused by external forces suggests that mechanical handling plays a significant role in model quality. Furthermore, the distribution of damage indicates distinct mechanisms of error formation - defects in upper models tend to spread across multiple regions, while those in lower models are more localized, emphasizing the need for targeted improvements in processing techniques.

From the perspective of student perception, survey results demonstrate a generally positive attitude toward digital technologies in dentistry, especially among students who view them as a competitive advantage for future practice. The data show that students who find 3D-printed models easy to use also tend to consider them a modern and beneficial tool for education. Moreover, those who see digital methods as useful are more likely to support the use of 3D virtual visualizations to enhance learning. These findings support the broader implementation of digital solutions in dental education, both to improve learning outcomes and to foster digital competencies in future dental professionals.

## Appendices

Appendix A 

**Table 6:** Questionnaire PDPD: Propedeutics of Children’s Dental Medicine; DM: Dental Medicine

No.	Question	Answers			
		A	B	C	D
Q1	Do you think you have gained basic knowledge about the digitization methods used in dental medicine?	Yes	No	I can't say	
Q2	Do you consider working with 3D printed models to be sufficiently modern?	Yes	No		
Q3	Did you find working with 3D printed models difficult?	No	Yes		
Q4	Which method is easier for you to work with?	On plaster models	On 3D printed models	I can't say	
Q5	Do you think that becoming familiar with digital methods in dental medicine would benefit you and make you more competitive after graduation?	Yes	No	I can't say	
Q6	Are you interested in learning more about digital working methods and their application in dental medicine?	Yes	No	I can't say	
Q7	Do you think there is a need for more comprehensive teaching of digitization methods in dental medicine?	Yes	No	I can't say	
Q8	Do you consider the teaching in the courses on PDPD and DM to be sufficiently modern?	Yes	No	I can't say	
Q9	Are you satisfied with the knowledge and skills you gain during lectures and practicals in PDPD and DM?	Yes	No	Yes, but they could be improved and updated	I can't say
Q10	Do you think the use of 3D virtual images in your training, which can be zoomed and rotated, would improve your understanding of the material by allowing you to view objects in three dimensions?	Yes	No	I can't say	
